# *In vitro* embryo production in small ruminants: what is still missing?

**DOI:** 10.1590/1984-3143-AR2023-0055

**Published:** 2023-11-10

**Authors:** Joanna Maria Gonçalves Souza-Fabjan, Gabriela Ramos Leal, Clara Ana Santos Monteiro, Ribrio Ivan Tavares Pereira Batista, Nathalia Oliveira Barbosa, Vicente José Figueirêdo Freitas

**Affiliations:** 1 Faculdade de Veterinária, Universidade Federal Fluminense, Niterói, RJ, Brasil; 2 Laboratório de Fisiologia e Controle da Reprodução, Faculdade de Veterinária, Universidade Estadual do Ceará, Fortaleza, CE, Brasil

**Keywords:** goat, IVM, IVC, sheep

## Abstract

*In vitro* embryo production (IVEP) is an extremely important tool for genetic improvement in livestock and it is the biotechnology that has grown the most recently. However, multiple ovulation followed by embryo transfer is still considered the leading biotechnology for embryo production in small ruminants. This review aimed to identify what is still missing for more efficient diffusion of IVEP in small ruminants, going through the IVEP steps and highlighting the main factors affecting the outcomes. Oocyte quality is essential for the success of IVEP and an aspect to be considered in small ruminants is their reproductive seasonality and strategies to mitigate the effect of season. The logistics for oocyte collection from live females is more complex than in cattle, and tools to simplify this collection system and/or to promote an alternative way of recovering oocytes may be an important point in this scenario. The heterogeneity of oocytes collected from growing follicles in live females or from ovaries collected from abattoirs remains a challenge, and there is a demand to standardize/homogenize the hormonal stimulatory protocols and IVM protocols for each source of oocytes. The use of sexed semen is technically possible, however the low market demand associated with the high costs of the sexing process prevents the routine use of this technique, but its higher availability is an important aspect aiming for greater dissemination of IVEP. New noninvasive approaches for embryo selection are key factors since the selection for transfer or cryopreservation is another difficulty faced among laboratories. Embryo selection is based on morphological traits, although these are not necessarily reliable in predicting pregnancy. Several issues described in this review must be considered by researchers in other to promote the diffusion of IVEP in small ruminants.

## Introduction

Small ruminants are important meat, milk, wool, and skin sources worldwide. However, a significant increase in demand for food is expected in the forthcoming years, and the current efficiency of small ruminant production systems is considered inadequate to meet global demands (Sargison, 2020). Regarding this aspect, reproductive biotechnologies are essential to increase productivity and accelerate the genetic gain of several production species (Paramio and Izquierdo, 2016; Souza-Fabjan et al., 2023). *In vitro* embryo production (IVEP) is the biotechnology that has grown the most in recent years, contributing to genetic improvement in livestock. Although the IVEP is well established in cattle, in small ruminants, multiple ovulation followed by embryo transfer (MOET) is still considered the leading biotechnology for embryo production (Fonseca et al., 2018).

**Figure 1:**
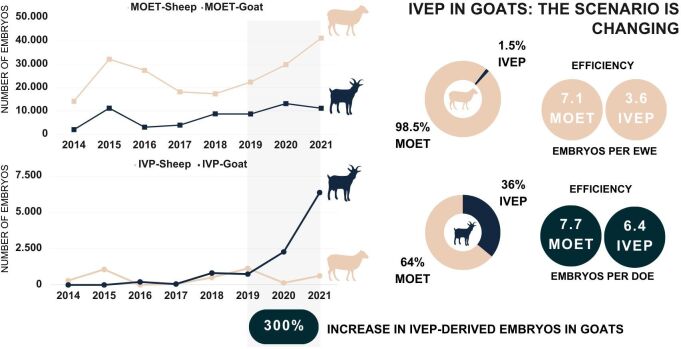
The figure reports the worldwide *in vivo* (MOET) and *in vitro* embryo production (IVEP) in small ruminants in the last years, based in the International Embryo Technology Society (IETS) newsletters published in 2022), highlighting the great increase in the number of IVEP-derived embryos in goats.

In the most recent newsletters released by the International Embryo Technology Society (Viana, 2022), it was evident that in sheep almost all embryos (98.5%) produced worldwide in 2021 were from traditional MOET, and this technique was twice as efficient (7.1 *vs* 3.6) for producing embryos per ewe than IVEP. In goats, however, the scenario has been changing (Table 1). Although MOET is still more predominant (64% vs 36%), the number of IVEP-derived embryos has increased by ~300% from 2019 to 2021 (Figure 1), and the efficiency of producing embryos per doe was quite similar in both techniques [7.7 (MOET) vs 6.4 (IVEP)]. The reasons why MOET remains the main technique for small ruminant embryo production are probably related to species particularities compared to cattle, costs involved, and outcomes (Souza-Fabjan et al., 2021).

**Table 1:** Overall *in vitro* embryo production efficiency outcomes in adult goats and sheep throughout time.

**Species**	**Years**	**MII** **(%)**	**Cleavage (%)**	**Blastocyst from cleaved (%)**	**Blastocyst from COC** **(%)**	**Reference**
Goat	1990-2000	44 - 97	17 - 80	0 - 45	0 - 30	Crozet et al. (1995); De Smedt et al. (1992); Le Gal et al. (1992); Izquierdo et al. (1998); Keskintepe et al. (1994); Keskintepe et al. (1998); Pawshe et al. (1996)
	2001-2010	55 - 84	26 - 83	10 - 72	5 - 47	Bormann et al. (2003); Herrick et al. (2004); Kątska-Książkiewicz et al. (2007); Koeman et al. (2003); Locatelli et al. (2008); Rho et al. (2001); Rodríguez-Dorta et al. (2007); Zhou et al. (2008)
	2011-2020	43 - 87	30 - 85	3 - 90	3 - 49	De et al. (2011); Widayati and Pangestu (2020); Romaguera et al. (2011); Souza et al. (2013); Fieni et al. (2012); Conceição et al. (2015); Veshkini et al. (2015); An et al. (2018); Pradeep et al. (2011); Kouamo and Kharche (2015); Wang et al. (2017)
	2021-Current	54 - 85	51 - 82	14 - 73	20 - 52	Ferreira-Silva et al. (2021); Souza-Fabjan et al. (2021); Shakir Hammoud and Adil Jebur (2022); Saini et al. (2022); Huang et al. (2022)
Sheep	1990-2000	76 - 100	26 - 92	15 - 51	13 - 31	O'Brien et al. (1997); Ledda et al. (1997); Gómez et al. (1998); Brown and Radziewic (1998); Ptak et al. (1999a); Ledda et al. (1999); Byrne et al. (2000)
	2001-2010	53 - 88	50 - 100	14 - 70	08 - 38	Cox and Alfaro (2007); Bai et al. (2008); Mossa et al. (2008); Shi et al. (2009); Wan et al. (2009); Modina et al. (2010); González et al. (2010); Shirazi et al. (2010)
	2011-2020	57 - 96	53 - 88	03 - 54	09 - 35	Catalá et al. (2012); Rose et al. (2013); Ghaffarilaleh et al. (2014); Leoni et al. (2015); Reader et al. (2015); Wang et al. (2016); Ariu et al. (2017); Masala et al. (2018); Cocero et al. (2019); Tian et al. (2019)
	2021-Current	59 - 92	28 - 84	14 - 33	03 - 39	Bebbere et al. (2021); Anzalone et al. (2021); Samereh et al. (2021); Fathi and Elkarmoty (2021); Sánchez-Ajofrín et al. (2022); Lorenzo-Torres et al. (2023)

MII: Metaphase II; COC: *Cumulus*-oocyte complex.

In general, the IVEP comprises four main steps: (1) the collection of *cumulus-*oocyte complexes (COCs); (2) COCs *in vitro* maturation (IVM); (3) *in vitro* fertilization (IVF), that is the co-cultivation of COCs and sperm; and (4) *in vitro* culture (IVC) or *in vitro* development (IVD) of the embryos until the blastocyst stage when they can be transferred to the uterus of synchronized female recipients (with pregnancy rates around 50%) or cryopreserved for future use, according to each production system (Cognié et al., 2004; Souza-Fabjan et al., 2021). For anatomical reasons, in small ruminants, the ovum pick-up is performed laparoscopically (LOPU). Despite it is considered a minimally invasive technique (Baldassarre, 2012; Souza-Fabjan et al., 2014b), performing LOPU is more expansive and complex than the bovine ovum pick-up (OPU; Souza-Fabjan et al., 2021), which certainly contributes to the lower use of IVEP in small ruminants.

Another aspect to be considered in small ruminants is their reproductive seasonality. It is well known that oocyte quality is essential for IVEP success (Kątska-Książkiewicz et al., 2007), being influenced by several factors, such as age, nutritional and health status, the hormonal protocol used for superstimulation, among others (Paramio and Izquierdo, 2016; Bragança et al., 2018). In small ruminants, however, the breeding season has a substantial impact on oocyte developmental competence, reflecting positively on IVEP results (Souza-Fabjan et al., 2021). Furthermore, despite advances in IVF, most protocols are currently based on concepts established many years ago, reinforcing the demand for optimizing the semen capacitation process to improve pregnancy rates (Cognié et al., 2004).

Although there are obviously some peculiarities and adaptations in each step, the results regarding IVEP rates in small ruminants are similar to those reported in cattle. It means that there is no reason for MOET to be still considered virtually the unique technique to produce embryos (Souza-Fabjan et al., 2021), and changes in the global scenario are expected, as experienced in goats recently. This review aims to describe the main factors affecting the outcomes in each IVEP step, in order to identify what is still missing for its wider dissemination in goats and sheep.

### Oocyte recovery

**Figure 2:**
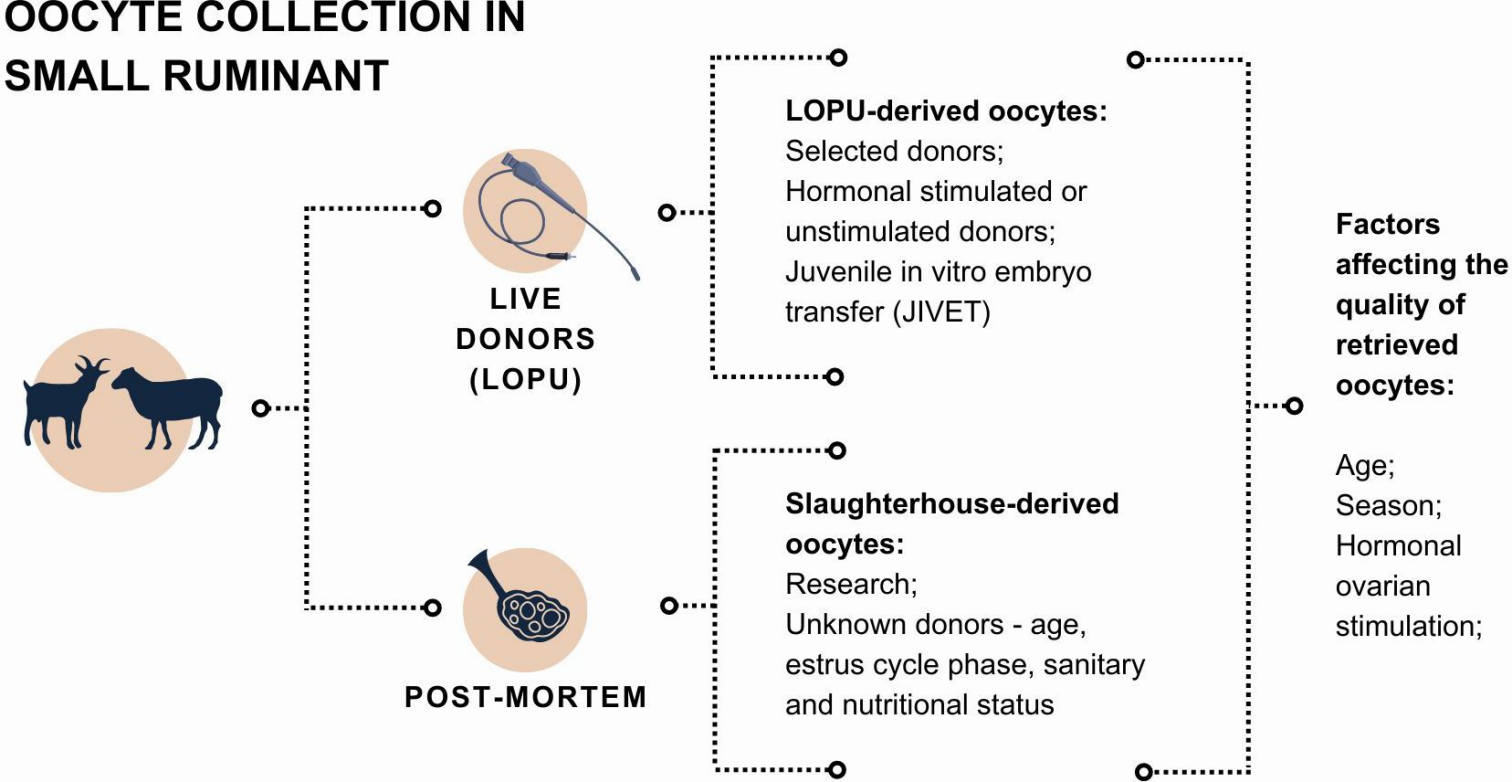
The figure illustrates the main sources of oocytes, the aspects related to each one as well as the factors affecting the quality of retrieved oocytes. LOPU (laparoscopic ovum pick up).

The first step of IVEP is the recovery of developmentally competent oocytes -defined as the oocyte’s ability to resume and achieve meiosis, be fertilized, develop into an embryo, and give rise to normal and fertile offspring (Mermillod et al., 2008). Oocyte recovery rates range from 35% (Alberio et al., 2002) to 90% (Cox and Alfaro, 2007) with an average of 10-14 oocytes/donor (Cox and Alfaro, 2007). For small ruminant IVEP, oocytes can be collected either *post-mortem* from slaughtered females or *in vivo* by laparotomy or laparoscopy (Figure 2).

### Post-mortem

The use of slaughterhouse-derived ovaries as a source of COCs allows the production of a high number of embryos of low cost, which are essential for research in the field of reproductive biology, including those studies aiming to improve IVEP outcomes (Paramio and Izquierdo, 2014; Souza-Fabjan et al., 2023). *Post-mortem*, the oocytes are commonly retrieved by either gently slicing the ovaries, puncturing, or dissecting follicles. The number of COCs recovered differs substantially, and it is known that the recovery technique can affect the COC quality (Majeed et al., 2019). Generally, slicing the ovarian tissue better preserves the COCs, while follicular aspiration may cause a loss in integrity of *cumulus* layers (Rodríguez et al., 2006); but the latter is the most used approach throughout different laboratories for recovering slaughterhouse-derived COCs. Although this type of source can also be used to recover gametes from high genetic value females that have been slaughtered to provide a last offspring (Tibary et al., 2005), in general, the *post-mortem* oocyte recovery presents limitations, such as the unknown stage of the estrous cycle as well as the age, sanitary, and nutritional status of slaughtered animals, factors that can affect the quality of embryos produced (Paramio and Izquierdo, 2014).

### In vivo

In live donors, COC recovery can be performed on selected females, resulting in an increased number of structures when females are hormonally stimulated. Thus, COCs can be collected by laparotomy, even though this method is practically unused due to the many advantages of laparoscopy (Falchi et al., 2022). The laparoscopic ovum pick-up (LOPU) was first described in sheep (Snyder and Dukelow, 1974) and has been used since then in small ruminants as the technique of choice (Avelar et al., 2012; Bragança et al., 2018).

The LOPU allows the visualization of ovaries and oocyte recovery by follicular aspiration through the use of a vacuum pump attached to a puncture needle (Souza-Fabjan et al., 2014a, b).

The aspiration device used in LOPU is important to respect the female tract integrity. It was demonstrated that LOPU can be safely repeated in the same animal up to 9-10 times in a year, without compromising the fertility of the donor, the number of aspirated follicles, oocyte recovery rates, and the quality of collected oocytes (Teixeira et al., 2011; Avelar et al., 2012). In addition, no complications such as fibrosis and adhesions were reported in sheep after repeated sessions of LOPU, reinforcing the minimally invasive nature of the procedure (Teixeira et al., 2011). Although the lower pregnancy rate of *in vitro*-produced embryos still limits LOPU widespread, it has been reported that the mimicry of the maternal environment *in vitro* can improve the viability of IVEP embryos (Rodríguez-Dorta et al., 2007).

Regarding oocyte recovery, an important factor to consider is the variability in the quality and number of oocytes collected. There are several points that can interfere in it, such as the female age, breed, season, stimulation treatment, and individual response to protocols, among others (Souza-Fabjan et al., 2021). Considering the aspects capable of playing a role in the outcomes, it is worth highlighting the main ones to understand how to optimize this important stage of the IVEP.

### Factors that affect the quality of retrieved oocytes

#### Age

It is well known that the age of the female donor affects the oocyte quality, as the developmental competence of COCs from prepubertal ewes (30-40 days old) is lower than those obtained from their adult counterparts (19.9 vs 51.3%) (Leoni et al., 2015). However, the use of prepubertal animals is an interesting tool as it reduces the interval between generations accelerating the genetic improvement of animals (Galli et al., 2001). Despite the presence of functional and ultrastructural deficiencies already reported in prepubertal goat oocytes (Paramio and Izquierdo, 2016) and their general lower competence (Ptak et al., 1999b; Leoni et al., 2015), Romaguera et al. (2011) demonstrated that goat COCs recovered from large follicles (≥3 mm) were similarly competent compared to those obtained from adult goats. On the other hand, aged females are associated with poor reproductive performance, probably due to their reduced ovarian reserve and impaired hormonal environment (Baldassarre et al., 2007).

#### Season

Reproductive seasonality has a substantial effect on oocyte quality (Mastromonaco and Gonzalez-Grajales, 2020). In adult goats, the season has a significant impact on the IVEP outcomes. The breeding season (autumn) leads to improved oocyte developmental competence generating a higher cleavage and blastocyst rate (Souza-Fabjan et al., 2021). In hormone-stimulated adult goats, the season appears not to affect the number of follicles aspirated and recovered oocytes (Pierson et al., 2004). However, regarding prepubertal goats, higher cleavage and blastocyst rates during the anestrous season (Catalá et al., 2018) were reported, suggesting that the photoperiod was not deleterious to this category.

In sheep, the season can influence the number and competence of recovered oocytes, but the cleavage rate tended to be higher in the anestrus season (Vázquez et al., 2010). Although the cleavage rate was similar during the breeding season, the blastocyst rate was higher when compared to the anestrus season (Mara et al., 2014). Interestingly, a subcutaneous implant of melatonin can improve the sheep oocyte developmental competence during the anestrus season (Vázquez et al., 2010), showing that approaches might be used to overcome potential seasonal effects.

#### Hormonal ovarian stimulation

The emergence of large antral follicles occurs in different waves during the reproductive cycle, being more frequently four in goats (Castro et al., 1999) and three in sheep (Evans et al., 2000). Selection of ovulatory follicle(s) may occur during the last wave and competent oocytes may be recovered from these follicles before atresia occurs. According to Kelly et al. (2005), oocytes are usually recovered from unstimulated or stimulated pre-pubertal donors (3-4 weeks to 5-6 months). Hormonal ovarian stimulation increases the number of follicles available to be aspirated and consequently, the number of recovered oocytes and, for this reason, is the first step of LOPU in goats and sheep (Gibbons et al., 2007; Souza-Fabjan et al., 2013; Souza-Fabjan et al., 2014a, b).

Several protocols have been proposed and tested for small ruminants, and usually involve the administration of FSH in either multiple doses (Baldassarre et al., 1996; Berlinguer et al., 2007; Sousa et al., 2011) or as a “one-shot” regimen associated with equine chorionic gonadotropin (eCG) (Baldassarre et al., 1996; Sousa et al., 2011), while its use in a single dose without the latter was ineffective (Armstrong et al., 1994; Baldassarre et al., 1996). Due to the ease of use in practical routine, the application of 80 mg FSH in a single dose with 300 IU eCG (“one-shot”) is often used in small ruminants (Baldassarre et al., 2002; Pierson et al., 2004; Sousa et al., 2011; Teixeira et al., 2011; Baldassarre, 2012; Santos et al., 2016).

**Figure 3:**
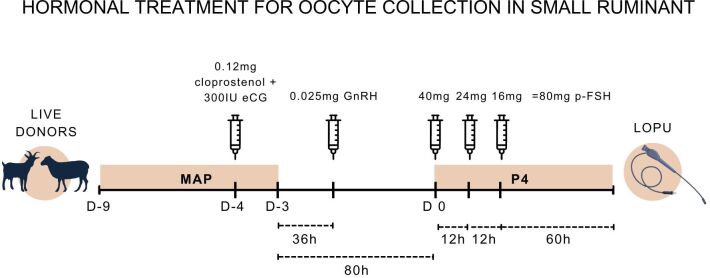
The schematic figure represents a hormonal protocol with progesterone and progestagen associated with multiple doses of FSH and a costing time of 60 h used for oocyte collection (Pinheiro et al., 2023). LOPU (laparoscopic ovum pick up); MAP (medroxyprogesterone acetate).

Recently, when 80 and 120 mg FSH were tested in both regimens of administration, 80 mg FSH was sufficient to stimulate the development of multiple follicles available for COC recovery in sheep, generating COCs with good morphology; however, the multiple-dose regimen was more indicated for producing COCs of potential better developmental competence (Bragança et al., 2018). A 12-h interval from the last FSH dose to the LOPU procedure (coasting time) has been successfully reported in sheep (Bragança et al., 2018, 2021b). However, recent evidence demonstrated that a 60-h interval of coasting (Figure 3) generated oocytes with a greater diameter, enhanced chromatin condensation pattern, and a better capacity to accumulate transcripts compared to the 12-h interval (Pinheiro et al., 2023).

For the hormonal stimulation treatments aforementioned described, there is always an association of gonadotropins with progestogens. During the treatment with gonadotropins, progesterone may be used to avoid ovulation through the regression of the dominant follicle caused by the inhibition of LH pulse (Greyling and van der Nest, 2000). Exogenous progesterone positively impacts oocyte competence (Bragança et al., 2021a), and has already been evidenced that progesterone treatment during follicular growth affects the oocyte developmental competence since progesterone concentrations can enhance the oocyte capacity to develop until the embryo stage, increasing embryo yield in MOET programs (Menchaca et al., 2018).

Regarding oocyte recovery, IVEP could be more efficient in small ruminants considering strategies to (i) mitigate the effect of season, (ii) simplify the LOPU system and/or promote an alternative way of recovering COCs in live females, and (iii) optimize and standardize/homogenize the hormonal stimulatory protocols.

### Oocyte selection and *in vitro* maturation (IVM)

Despite constant advances in IVM studies (Souza-Fabjan et al., 2014a; Leal et al., 2018), the search for optimal conditions for IVM continues in high demand, since mimicking *in vitro* the processes that occur during oocyte maturation *in vivo* is still a challenge (Albuz et al., 2010; Leal et al., 2018; Leal et al., 2022). The selection of high-quality COCs is fundamental to IVEP success and it depends on many intrinsic and extrinsic factors (Kątska-Książkiewicz et al., 2007; Souza-Fabjan et al., 2021). The oocyte sample can be heterogeneous, leading to different outcomes. In addition, it was already established that despite the LOPU-derived oocytes and slaughterhouse-derived oocytes might present a similar intrinsic quality, they may have different IVM kinetics and requirements (Souza-Fabjan et al., 2014a, 2016, 2021).

#### Oocyte selection

After collection, high-quality oocytes are selected for IVM. The selection aims to screen the recovered oocytes and choose those with the greatest developmental competence, defined as the oocyte capacity to successfully complete nuclear and cytoplasmic maturation, undergo fertilization, develop to the blastocyst stage, induce pregnancy and birth of living offspring (Sirard et al., 2006).

Different parameters can be used to identify the oocyte developmental competence potential, such as the size of the follicle from which the oocyte is collected, and the oocyte diameter since there is a positive correlation between follicle size and oocyte diameter, among others (Paramio and Izquierdo, 2016). But, in general, oocytes are selected based on morphological characteristics of their ooplasm and *cumulus* cells, and according to this selection, they can be graded from 1 to 4 (Almeida et al., 2011): I) finely granulated oocyte cytoplasm and multilayered compact *cumulus* cells; II) same oocyte cytoplasm as grade I and one to three layers of *cumulus* cells; III) heterogeneous oocyte cytoplasm and incomplete or no cellular vestment; IV) oocyte with abnormal shape and heterogeneous oocyte cytoplasm or apoptotic oocytes in jelly-like c*umulus*-corona cells (considered degenerate). Small ruminant oocytes can have slightly different graduations (Souza-Fabjan et al., 2016), however, although the morphological evaluation is still considered the main method of oocyte selection for IVEP, it is a subjective procedure that does not strongly predict oocyte competence (Almeida et al., 2011).

For this reason, the combination of morphological selection with brilliant cresyl blue (BCB) staining is a strategy that can be used to screen oocytes with better developmental competence (Bragança et al., 2021b). This staining categorizes the evaluated oocytes into two groups: BCB+, which have greater developmental competence, and BCB -, which in turn, presents a lower developmental competence (Paramio and Izquierdo, 2016; Souza-Fabjan et al., 2021). This categorization is based on the glucose-6-phosphate dehydrogenase enzyme activity in reducing BCB. The activity of this enzyme is usually high during oocyte growth (oocytes with a not stained cytoplasm; BCB-) and low at the end of growth (oocytes with a blue-stained cytoplasm; BCB+) (Rodríguez-González et al., 2002). This staining has been substantially used in small ruminants (Catalá et al., 2012; Bragança et al., 2018) to select a more homogeneous oocyte pool regarding developmental competence for IVEP.

It is worth mentioning that during oocyte collection, the follicular aspiration can lead to the loss of *cumulus* cells and those oocytes that became denuded due to the pressure of the aspiration system will not be selected, since the *cumulus* cells are related to the fertilization rate in most species (Zhang et al., 1995; Suzuki et al., 2000) and, morphologically, they cannot be differentiated from those from atretic follicles. However, it was already established that some oocytes that are already denuded at collection can develop until the blastocyst stage if they are submitted to IVM and IVF in the presence of intact COCs (Souza et al., 2013). This tool might represent an additional source of embryos in valuable females.

#### Oocyte maturation

Oocyte maturation is a delicate process in which the oocyte undergoes several molecular and structural changes and acquires the intrinsic competence to support fertilization and the embryo’s early development until major activation of the embryonic genome (Meirelles et al., 2004; Ferreira et al., 2009; Leal et al., 2018). Indeed, oocyte maturation includes the resumption of meiosis and its progression to the stage of metaphase II (nuclear maturation), also protein synthesis, molecular changes, and redistribution of intracellular organelles (cytoplasmic maturation) (Leal et al., 2018).

After oocyte selection, groups of approximately 50 oocytes are incubated in four-well plates in 500 µL of the medium, under 5% CO_2_ in air at 38.5-39 ºC, with maximum humidity, for 22-24 h (Souza-Fabjan et al., 2019). The IVM medium must contain the necessary components to enable the oocytes to complete nuclear and cytoplasmic maturation, which are distinct but connected events (Ferreira et al., 2009; Leal et al., 2018). Despite many advances in IVEP throughout the years, the IVM efficiency is still lower than *in vivo* maturation, since mimicking *in vitro* the optimal conditions for maturation is still one of the main obstacles for IVEP (Leal et al., 2018). Thus, the intrinsic quality of immature oocytes and also the IVM conditions are important to ensure the success and efficacy of IVEP and quality (Souza-Fabjan et al., 2014a).

#### IVM media

TCM-199 (Tissue Culture Medium - 199) is used as an IVM base medium in small ruminants in most laboratories (Souza-Fabjan et al., 2021). It is often supplemented with hormones, antibiotics, energy, and protein sources. The hormonal supplementation most commonly used are FSH (porcine or ovine), LH, and/or 17b-estradiol (Cognié et al., 2004; Paramio and Izquierdo, 2016; Zhu et al., 2018). Although estradiol is routinely added to IVM media in these species, its use seems quite controversial (Cognié et al., 2004). In general, it seems beneficial under defined conditions but has no role when the media already has a biological fluid such as follicular fluid (FF) or fetal calf serum (FCS; Guler et al., 2000).

The FCS, FF, estrus sheep or goat serum, and bovine serum albumin (BSA) are the most used protein sources for IVM (Cognié et al., 2004), however, these are complex substances that can make reproducibility difficult due to chemical variations. The beneficial effect of FCS (Shabankareh and Zandi, 2010), for example, is due to the presence of substances such as growth factors, vitamins, hormones, vitamins, lipids, and proteins that influence the maturation process but the variation of its components between batches limits the establishment of a defined medium in addition of being associated with sanitary risk (Souza-Fabjan et al., 2019, 2021). FF from large follicles (>4 mm) also can be used as a supplement in IVM and presents a positive effect on maturation when it is recovered from gonadotropin-stimulated follicles (Cognié and Poulin, 2000). In general, TCM can be used to provide a different IVM media system according to the protein source supplementation: supplemented TCM with different types of serum creates an undefined system, with BSA generating a semi-defined system and with totally purified molecules leading to a defined system, which has been the worldwide trend (Souza-Fabjan et al., 2021). It was already demonstrated that a simplified maturation medium containing only TCM-199 supplemented with epidermal growth factor (EGF) and cysteamine can be efficiently used and the supplementation with FCS or BSA did not bring any extra benefits for slaughterhouse-derived COCs (Rodríguez-Dorta et al., 2007; Souza-Fabjan et al., 2014a).

Interestingly, after parthenogenetic activation, similar blastocyst rates were detected between LOPU- and slaughterhouse-derived oocytes, suggesting that the oocytes from both sources may have similar intrinsic quality (Souza-Fabjan et al., 2014a, 2016). However, oocytes from different sources (LOPU and slaughterhouse) present different IVM kinetics and requirements, and a more complex media is necessary to achieve success in this step (Souza-Fabjan et al., 2013; Souza-Fabjan et al., 2014a, 2016). Even though we previously demonstrated that slaughterhouse-derived oocytes mature faster than the LOPU ones depending on the IVM medium, a reliable and simpler strategy may be the use of 22 h of IVM for COCs derived from both sources (Souza-Fabjan et al., 2019, 2021).

#### Strategies to improve IVM

Since the optimal conditions for oocyte IVM have not yet been established, many strategies have been adopted and remain the focus of many studies (Leal et al., 2018). Considering that the oxygen tension used on IVM (approximately 20% O_2_) can potentiate the formation of reactive oxygen species (ROS), which can compromise cellular functioning, the use of antioxidants is an important tool to improve IVM. When added to the IVM media, resveratrol (1 mM) improved the blastocyst rate (Piras et al., 2019), crocetin (1 µM) reduced ROS levels (Menéndez-Blanco et al., 2020), both studies in prepubertal goats, while cysteamine (100 µM) enhanced the blastocyst rates in adult goats (Cognié et al., 2003).

Another important aspect related to the IVM low efficiency is the precocious oocyte meiotic resumption following the artificial removal of COCs from the antral follicles. Oocytes can resume meiosis after it, but the early occurrence of nuclear maturation leads to a loss of synchrony with cytoplasmic maturation (Gilchrist and Thompson, 2007; Leal et al., 2018). In this sense, forskolin (100 mM) and isobutyl-1-methylxanthine (500 mM), substances that can modulate the pathways involved in the reestablishment between cytoplasmic and nuclear maturation, were already added to the IVM media of small ruminants (Leal et al., 2022) with positive results regarding embryo production in goats (Suresh et al., 2021) and embryo quality in sheep (Rose et al., 2013).

**Figure 4:**
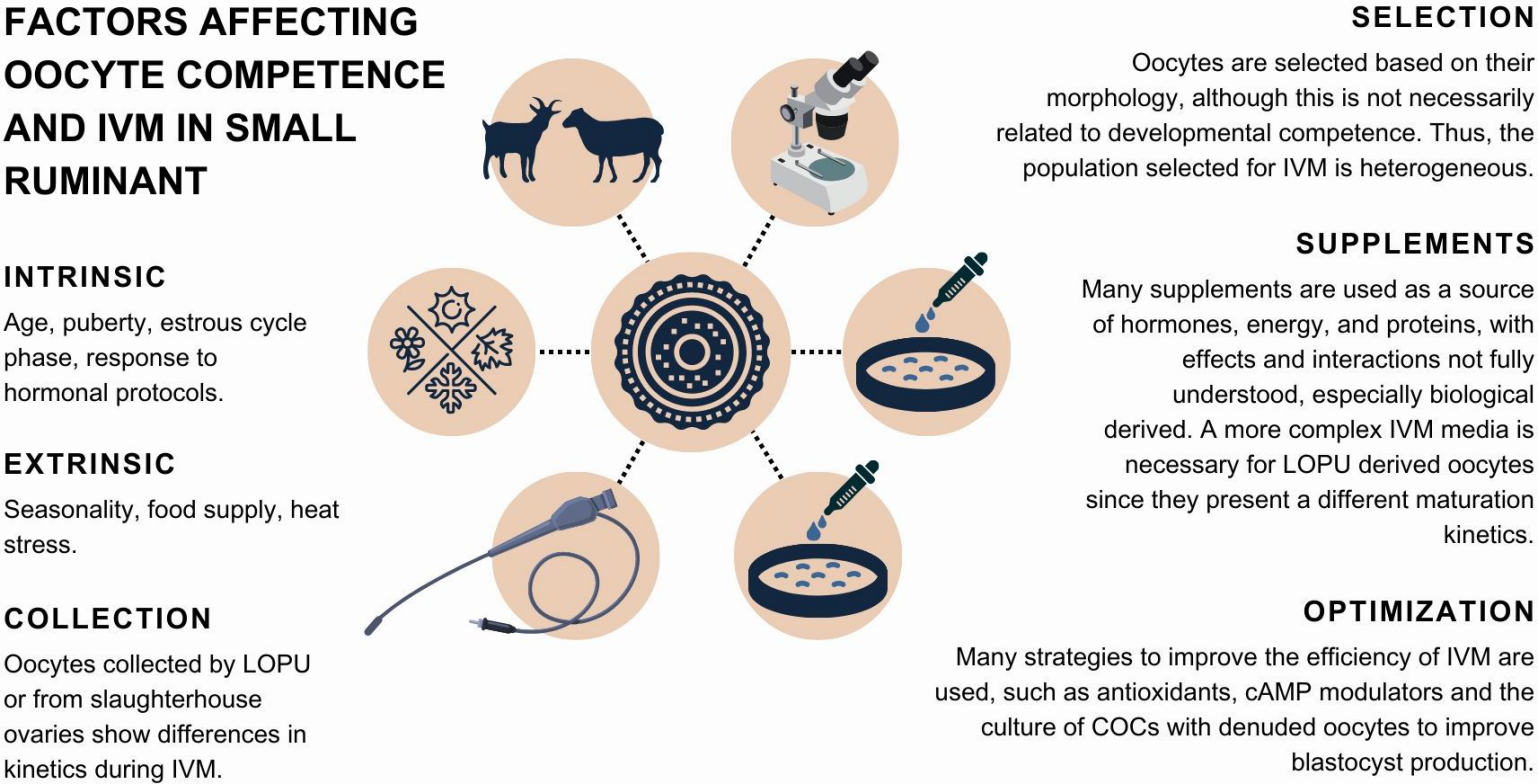
The image describes the factors that influence oocyte competence and the supplements and strategies used in IVM to improve it. IVM *(in vitro* maturation).

Studies using insulin-transferrin-selenium and l-ascorbic acid on IVM were already performed, and although they presented no impact on the blastocyst yield in goats (Hammami et al., 2013) and sheep (Catalá et al., 2013), in goat, embryos had a higher quality. Strategies to improve IVEP through IVM are still under development. The procedures in different laboratories are quite similar, with minor modifications, however, the results still present a significant variation (Paramio and Izquierdo, 2016), and for this reason, a standardization of IVM protocol/system appropriate to each source/type of COC, for example, is an important missing point in the search for better results (Figure 4).

### *In vitro* fertilization (IVF)

Current protocols for IVF in small ruminants are based on concepts established more than 25 years ago, where oocytes in metaphase II are co-incubated with previously selected and capacitated sperm, usually for a period of 12-18 h (Souza-Fabjan et al., 2023). Although this approach maximizes the number of fertilized oocytes, it exposes the presumptive zygotes to a high concentration of ROS generated by active and degenerated sperm, in addition to positively correlating with polyspermy. To mitigate these effects, some strategies have been adopted during IVF, mainly aimed at increasing embryonic quality. Below we will describe the main ones currently employed for optimizing the efficiency of each stage of IVF.

#### Sperm selection

Specifically in small ruminants, when it comes to frozen-thawed semen, the method of choice for sperm selection is the Percoll Gradient (45%/90%), due to the greater efficiency in the sperm recovery rate (Garcia-Garcia et al., 2007; Wan et al., 2009; Wang et al., 2013; Souza-Fabjan et al., 2016; Souza-Fabjan et al., 2021; Bragança et al., 2021a). As a result, most studies using sexed semen adopt density gradients for sperm selection (Hollinshead et al., 2004; de Graaf et al., 2007; Bathgate et al., 2013). Alternatively, the swim-up technique can also be used, especially for fresh semen (Romaguera et al., 2011; Hammami et al., 2013; Shirazi and Motaghi, 2013). When frozen-thawed semen was selected by the two methods, in addition to greater efficiency of sperm recovery, a higher rate of pronuclear formation was also observed in the group of oocytes co-cultured with spermatozoa selected by the Percoll Gradient (Rho et al., 2001). This result can be attributed to the beneficial effect of the centrifugation process in the removal of disabling factors, such as proteins and other substances that coat the sperm membrane and prevent capacitation.

#### Sperm capacitation

*In vitro* induction of sperm capacitation in small ruminants can be achieved by exposing sperm cells to capacitating agents for 15-60 min prior to co-culture with COCs. Among the main sperm enablers, we can highlight estrus goat or sheep serum (ESS), a mix of penicillamine, hypotaurine, epinephrine (PHE), heparin, and ionomycin (Souza-Fabjan et al., 2021). In addition to those classical capacitation inducers, the set of studies carried out by the Muiño-Blanco group demonstrates that cyclic adenosine monophosphate (cAMP) agonists can induce sperm capacitation via tyrosine protein phosphorylation (Grasa et al., 2006; Colas et al., 2008), which qualifies them as an alternative for inducing sheep semen capacitation. Our laboratory routinely uses the combination of ESS (10%) and heparin (5 µg/mL) during the co-cultivation of male and female gametes, with a blastocyst production efficiency of 54%, compared to 42% when only ESS was used (Souza et al., 2013). However, another research group demonstrated that in comparison to 2% ESS, the use of 10% ESS resulted in sheep blastocysts presenting a greater abundance of transcription involved in apoptosis and antioxidant defense (Sánchez-Ajofrín et al., 2022). These data suggest that a high concentration of ESS during IVF may influence the quality of sheep embryos at later stages of development. This concept of reducing or even replacing serum in IVEP media has been an important trend aiming to improve embryo quality.

#### Gamete co-cultivation

Usually, sheep IVEP laboratories use synthetic oviduct fluid (SOF) for gametes co-culture (Bai et al., 2008; Wang et al., 2013), while Tyrode's albumin lactate medium pyruvate (TALP) is more often used for goats (Kątska-Książkiewicz et al., 2007; Han et al., 2008; Hammami et al., 2013). Under these conditions, data previously compiled by our group suggest a higher incidence of polyspermy in both adults (16-47% vs. 3-21%) and prepubescent (0.5-39% vs. 5-26%) goats compared to sheep, respectively (Souza-Fabjan et al., 2021). These results could be explained by the difference in the gamete co-culture medium during IVF. However, when using SOF medium for goat IVF, we also observed a high rate of polyspermy (28-37%), despite high cleavage (72-88%) and blastocyst (27-51%) rates (Souza et al., 2013; Souza-Fabjan et al., 2014a; Souza-Fabjan et al., 2016). Aiming at a better mimicry of the oviductal environment during IVF, we evaluated the effect of replacing ESS with oviduct fluid on the production efficiency of monospermic zygotes. For this, IVM-oocytes were co-cultured with spermatozoa (1.0, 2.0, or 4.0 x 10^6^ cells/mL) for 18 h in SOF medium supplemented with 5 μg/mL heparin and 10% ESS (groups CTRL1, CTRL2 and CTRL4) or 10% oviduct fluid (groups OF1, OF2 and OF4) obtained from anestrus goats. When data were plotted independently of concentration, a beneficial effect of oviduct fluid supplementation on the production efficiency of monospermic zygotes was observed. However, this effect did not affect the rates of cleavage, blastocyst, hatching, and number of cells per blastocyst, implying that polyspermic embryos can develop up to blastocyst (Bragança et al., 2021a). This was previously reported in the swine species by Han et al. (1999), who demonstrated that polyspermic porcine zygotes can develop with the same competence as normally fertilized zygotes.

To reduce the negative effect of prolonged exposure of presumptive zygotes to high concentrations of ROS and the incidence of polyspermy in sheep, Anzalone et al. (2021) assessed oocytes/presumptive zygotes co-cultured with spermatozoa every 30 min to determine the time required for fertilization. After consistent 15 replicates, the authors concluded that 4 h of gamete interaction is sufficient for successful fertilization. Not surprisingly, a higher polyspermy rate was seen in conventional IVF (16 h, 17.8%) compared to short IVF (4 h, 6.5%), using the sperm concentration of 5 x 10^6^ cells/mL, whilst the latter led to higher cleavage rate, blastocyst production, and total cell number. Overall, these authors showed that a shorter oocyte-sperm incubation increases the efficiency and quality of blastocyst production *in vitro*, probably as a consequence of a shorter exposure of the presumptive zygotes to ROS and a reduction in the rate of polyspermy. At physiological levels, ROS play important roles in male fertility, as they regulate many events related to the acquisition of sperm fertilizing capacity, such as hyperactivation and phosphorylation involved in sperm capacitation (de Lamirande and Gagnon, 1995). However, high levels of ROS can react with numerous biological molecules, such as lipids, proteins, and DNA, triggering rapid chain reactions and irreversible damage to DNA and cells. Additionally, the open and highly accessible conformation of the embryo's genome during the pronuclear phase and early development makes them more vulnerable to ROS. Corroborating this hypothesis, Leoni et al. (2007) demonstrated that a low (5%) O_2_ atmosphere during IVF positively affected the production of high-quality sheep blastocysts when compared to 20% O_2_.

Although fertilization is possible without *cumulus* cells (CCs), their presence around the oocyte at the time of fertilization appears to increase the chance of successful fertilization at least *in vitro* (Toyoda et al., 1993; Chian et al., 1995; Van Soom et al., 2002). In small ruminants, Souza et al. (2013) and Santos-Neto et al. (2020) demonstrated a beneficial effect of CCs during goat and sheep IVF, respectively. In the former, regardless of intimate contact with the oocyte or free in the well, the presence of CCs enhanced the blastocyst yield, raising a hypothesis that beyond their role as a physical barrier in polyspermy control, they may exert an additional benefit in the gamete interaction. In this sense, Vandevoort et al. (1997) demonstrated that pre-incubation of gametes with hyaluronic acid can increase the ability of the zona pellucida to induce the acrosomal reaction of monkey sperm. In some animals, progesterone secreted by CCs can trigger the acrosome reaction of sperm (Yanagimachi, 2022).

#### Limitations of commercial use of sexed semen in small ruminants

**Figure 5:**
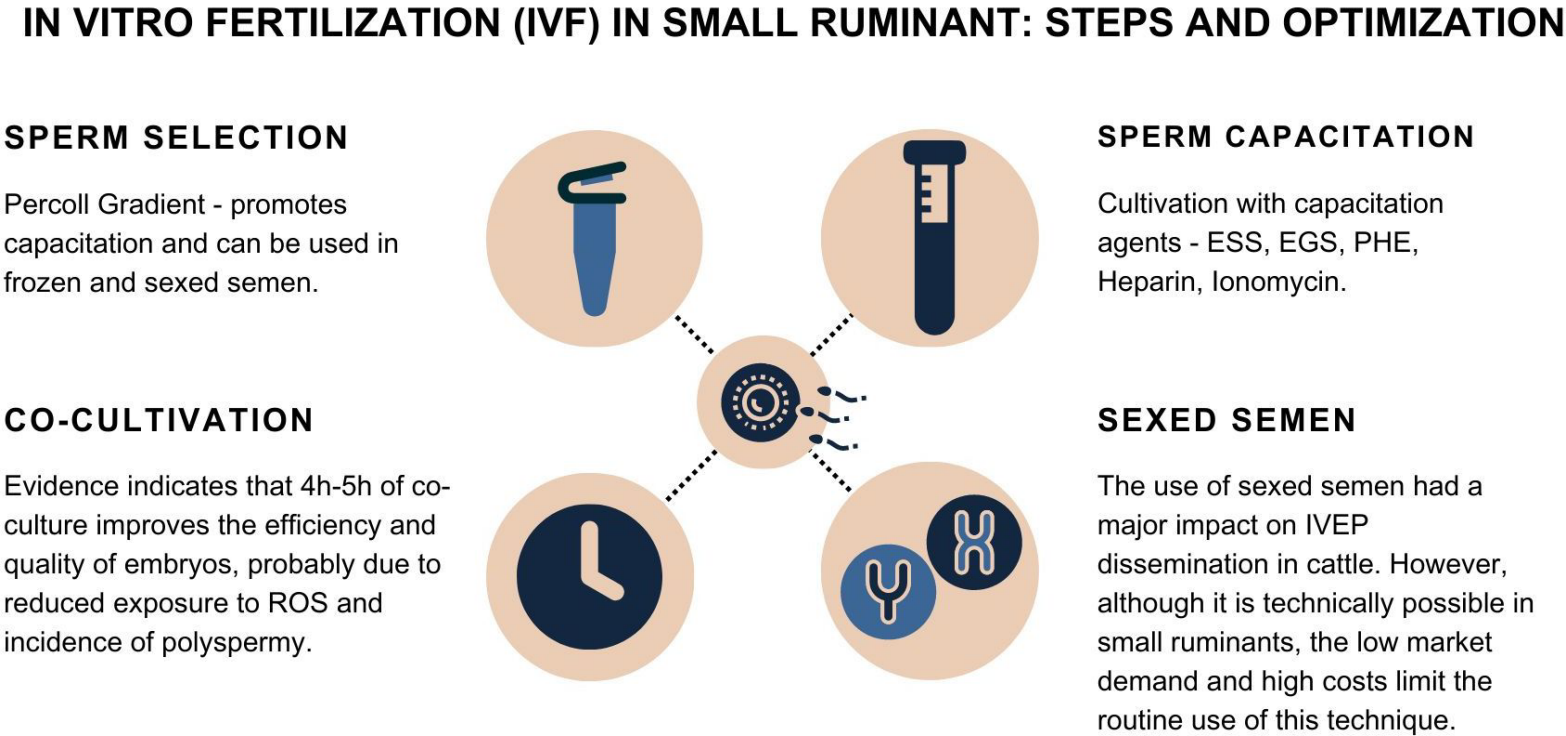
The image presents the main steps of IVF, limitations, and strategies to improve this step of IVEP. ESS: estrus sheep serum; EGS: estrus goat serum; PHE: epinephrine.

The combination of IVF with sexed semen is an extremely important alternative to boost the commercial use of IVEP in small ruminants. The flow cytometer technology currently used to classify the population of spermatozoa carrying X and Y chromosomes makes it possible to identify and select individual sperm. This selection, based on the substantial difference in DNA content between the X and Y chromosomes in sperm, makes it possible to clearly classify these two populations with an accuracy of about 90% (Parrilla et al., 2004). Analysis of the IVF capacity of frozen-thawed ram semen before and after sexing demonstrates its viability not only in IVEP but also in offspring after embryo transfer (Hollinshead et al., 2004; de Graaf et al., 2007). Of note, in goats, only 10 years ago the spermatozoa were sex-sorted by flow cytometry, frozen, and further used to produce offspring (Bathgate et al., 2013). Regarding the molecular aspects, Beilby et al. (2011) demonstrated that flow cytometry sexing procedures do not compromise embryo IVD, or the expression of genes associated with epigenetic alterations (DNA methyltransferase 3a - DNMT3; variegation suppressor 3-9 homolog 1 - SUV39H1), cellular stress (heat shock protein 70 - HSP70), oxidative stress (glucose-6-phosphate dehydrogenase - G6PD) and cellular metabolism (solute transporter family 2 member 3 - SLC2A3). However, although the use of sexed semen is technically possible in small ruminants, the low market demand associated with the high costs of the sexing process prevents its routine use. In this sense, higher availability of sexed semen in different countries and better control of polyspermy are important aspects to be considered in the optimization of the IVF stage as relevant points for greater IVEP dissemination (Figure 5).

### *In vitro* development (IVD)

**Figure 6:**
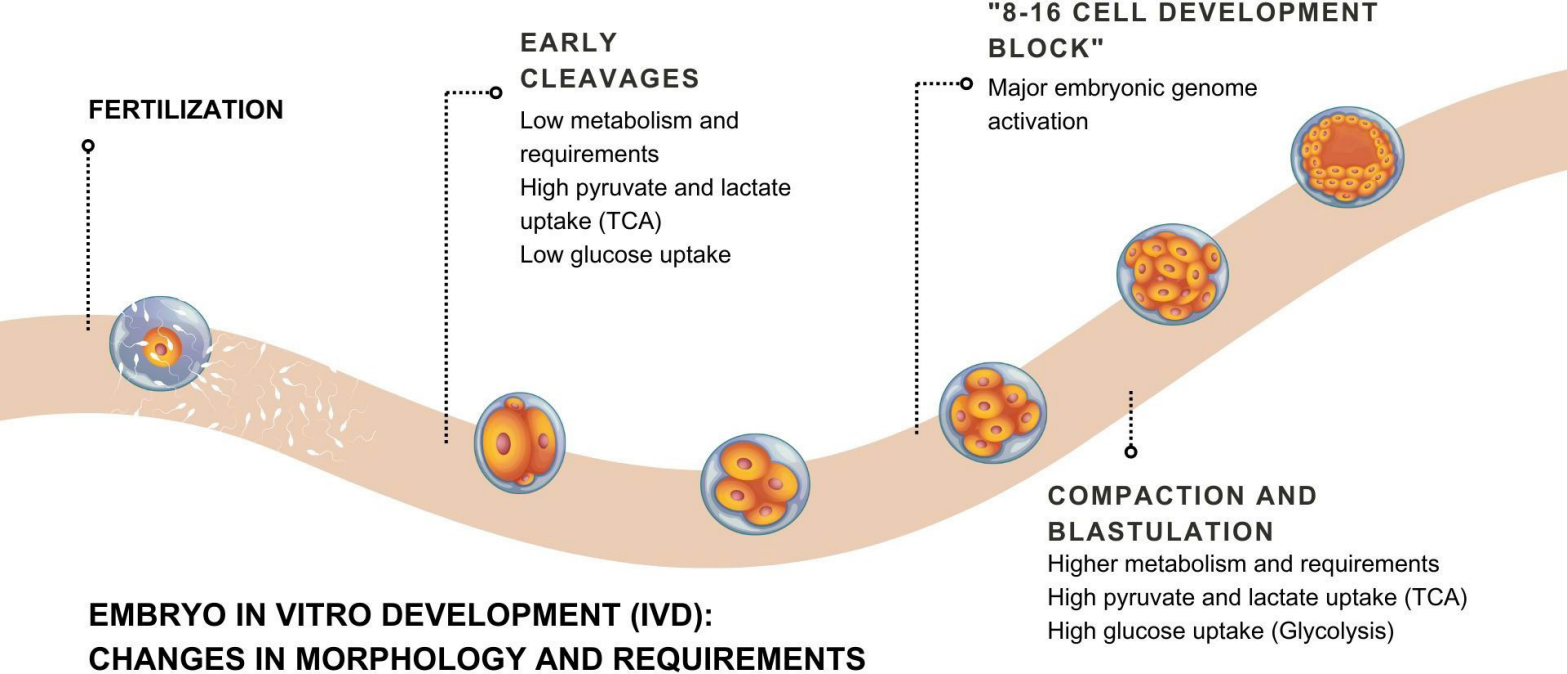
Schematic figure illustrating the changes that occur during *in vitro* embryonic development.

After fertilization, presumptive zygotes are transferred to the next and final step of IVEP - embryo *in vitro* culture or development (IVD). The IVD lasts 6 to 8 days in which fully expanded 100-cell blastocysts will develop from a single cell. Usually, the cleavage rate is evaluated 48 h after IVF, and blastocysts are evaluated on days 7 or 8 of IVD (Souza-Fabjan et al., 2013; Souza- Fabjan et al., 2014a; Bragança et al., 2021a). Morphologically, the embryos undergo cleavage, compaction, and cavitation, while on a cellular and molecular level, changes are much more complex and not fully understood, such as the mechanisms involving maternal to embryonic transition and energy metabolism (Figure 6). The purpose of IVD is to provide appropriate conditions for embryos to develop, as occurs in the oviduct, including osmolality, ion composition, temperature, pH, CO_2_ and oxygen levels, carbohydrates, amino acids, lipids, fatty acids, proteins, growth factors, and cytokines. However, researchers still struggle to offer optimal conditions for embryo development, as 50-60% of the presumptive zygotes fail to develop into the blastocyst stage. The “8- to 16-cell developmental block” is a critical culture-induced event often observed in ruminant IVD embryos that result in embryonic death and corresponds to the period of major embryonic genome activation (Telford et al., 1990). In addition to the lack of understanding about the specific requirements and mechanisms by which embryos can develop properly, other factors affect the IVEP propagation, such as the inconsistency in results between different laboratories and the low viability of obtained embryos.

#### Embryo IVD medium

Among the pioneering studies on IVD embryos requirements, Tervit et al. (1972) described the SOF - a media based on the biochemical analysis of sheep oviduct fluid, that combined with low oxygen tension, successfully supported the development of sheep and cattle embryos through the “8- to 16-cell developmental block” until the blastocyst stage. However, results were few and highly variable and another strategy emerged - the cell co-culture. Gandolfi and Moor (1987) demonstrated that the co-culture of presumptive zygotes with oviductal epithelial cells (OEC) was able to support the development through the “8- to 16-cell developmental block” and the viability of obtained embryos was comparable to those cultured *in vivo*, in recipient ewes. As the interaction between the embryo and the reproductive tract was further investigated, it became clear that the somatic cells in the culture contributed to the production of growth factors (EGF, tumor growth factors alpha and beta1) and the removal of inhibitory components from the culture medium, such as free radicals, cell metabolites and ammonia (Thompson, 1996, 2000). Rodríguez-Dorta et al. (2007) tested the effect of goat oviduct epithelial cells (GOEC) co-culture and observed that although the culture with SOF alone yielded a higher blastocyst rate compared to GOEC co-culture (28 vs 20%), after transfer of vitrified-thawed embryos, the latter yielded higher rates of pregnancy (56 vs 14%) and offspring born (33 vs 9%) than the former. The strategy of co-culture provided significant advances in the application of IVEP, but not in terms of knowledge on IVD embryo requirements, as it is not always possible to identify the components that are being secreted by somatic cells in co-culture and concentrations of the components change according to the physiological state of the cells, so the results are variable and difficult to predict (Bavister, 1995).

Other strategies to improve IVD embryo development employed biologically derived supplements such as FCS and BSA, known as undefined and semi-defined culture systems, respectively. The use of FCS during embryo culture has been extensively studied and is related to a long list of unwanted effects on embryo development and cryotolerance (Rizos et al., 2003). A set of changes in growth after transfer of embryos cultured with serum, known as “large offspring syndrome”, has been observed in different species, including small ruminants (Thompson et al., 1995; Sinclair et al., 1999). However, FCS is still widely used in most laboratories due to its potent effect on promoting embryo production (Hagemann et al., 1998). BSA is another supplement widely used in IVD, as albumin is the main protein found in the reproductive tract of mammals, with significant role in embryo nutrition, especially after compaction (Dunglison and Kaye, 1993; Dunglison et al., 1995). Garcia-Garcia et al. (2007) compared the SOF supplementation with BSA and 5% FCS and blastocyst rates were similar (22% vs 24% respectively); however, the blastocysts derived from the FCS system presented higher hatching capacity compared to BSA (44% vs 87%). Other studies replaced serum by BSA and amino acids in SOF media and found mean birth weight and incidence of abnormalities similar to those derived from *in vivo* counterparts (Thompson et al., 1995; Sinclair et al., 1997).

Currently, in most laboratories, embryos are cultured in semi-defined media with BSA, little or no FCS, in the absence of cell co-culture and at low oxygen tension. However, due to the undefined and variable composition, undesirable effects on embryo development and the risk of biological contamination, efforts have been directed towards defined culture media (Bavister, 1995). Unlike biological derived media, the use of defined culture media enables to identify and understand components that might influence embryo development. Polyvinyl alcohol (PVA) is a possible substitute, due to an albumin-like surfactant activity. However, evidence suggests that blastocysts cultured in PVA-supplemented media have an altered metabolic profile compared to those cultured in the presence of albumin or produced *in vivo* (Thompson et al., 1998). Studies in goats (Koeman et al., 2003) and sheep (Garcia-Garcia et al., 2007) have demonstrated that the IVD in defined media can support embryo development into the blastocyst stage, although it could compromise hatching rates (Garcia-Garcia et al., 2007). So is still not clear whether and which protein source can replace albumin during IVD.

Another trend in IVD is the use of sequential media systems (Gardner and Lane, 1997), based on evidence that the embryo nutritional requirements change as it moves towards the uterus. The energy metabolism is considered a key regulator of embryo development and its demand and consequently production increases as the embryo develop - at early cleavage stages is low and ATP production is based predominantly on pyruvate and lactate consumption by the TCA-cycle. Also, during early stages the glucose uptake is low, but as the embryo develops towards compaction and blastulation the ATP demand increases, causing a significant increase in glucose consumption and glycolysis contribution to ATP production (Thompson, 2000).

#### Embryo quality

Evidence in cattle suggest that the key factors for IVEP success, in terms of production rate, are the intrinsic oocyte quality and IVM conditions, while for embryo quality, considering viability post-transfer and cryotolerance, are the culture conditions (Rizos et al., 2002). So, as a result of sub-optimal developmental conditions, IVEP-derived embryos have lower viability compared to *in vivo* counterparts. Several studies demonstrate culture-induced modifications in IVD bovine embryos, such as cytoplasm lipid accumulation (Melo-Sterza and Poehland, 2021), fragile zona pellucida (Cannon and Menino, 1998), higher chromosomal abnormality (Slimane et al., 2000).

**Figure 7:**
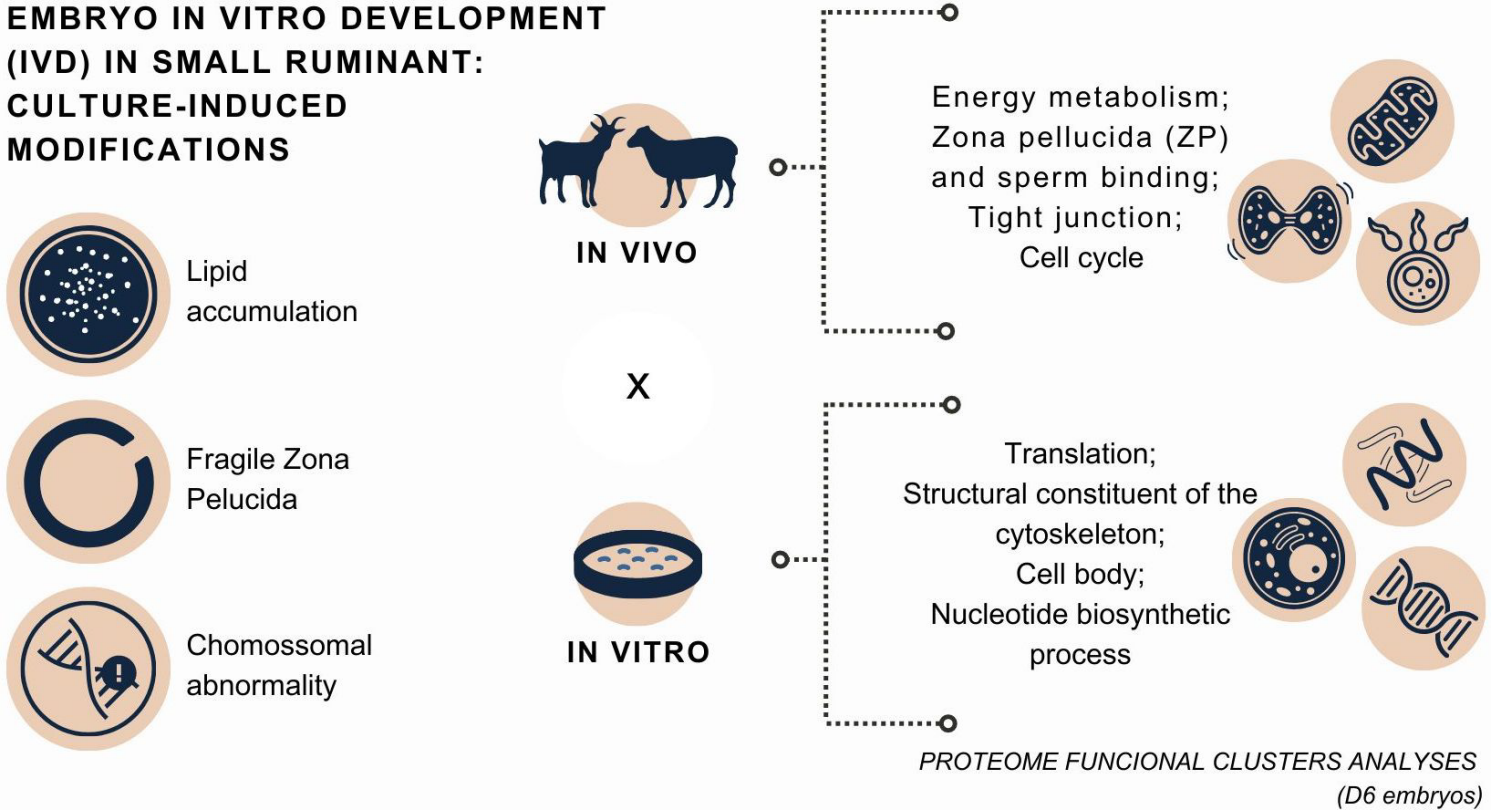
The schematic figure shows characteristics of different patterns of *in vivo* or *in vitro* produced embryos.

Recently, proteome studies revealed differences in functional clusters analyses between sheep embryos (day 6) produced *in vitro* and *in vivo*. Authors observed that the functional clusters of IVD embryos proteome are related to translation, structural constituent of ribosomes, ribosomes, nucleosomes, structural constituent of the cytoskeleton, microtubule-based process, translation initiation factor activity, regulation of translational initiation, cell body and nucleotide biosynthetic process (Passos et al., 2022). Conversely, *in vivo* derived embryos functional clusters are associated with energy metabolism (TCA cycle, pyruvate, and glycolysis metabolism), zona pellucida (ZP), MAPK signaling pathway, tight junction, binding of sperm to ZP, translation, proteasome, cell cycle and calcium/phospholipid binding proteins (Sanchez et al., 2021). Such studies have great potential to elucidate physiological mechanisms related to embryo development and culture-induced modifications (Figure 7).

The selection of embryos for transfer or cryopreservation is another difficulty encountered by laboratories. Currently, selection is based on morphological traits and quality is used evaluated according to the Manual of the International Embryo Transfer Society (IETS). Although morphological selection is not necessarily reliable compared to omics technologies (Krisher et al., 2015), embryo metabolism (Thompson et al., 2016; Nõmm et al., 2019), or developmental kinetics (McLennan et al., 2020; Fryc et al., 2021). Despite recent advances, there is still a demand for selection methods that are reliable, non-invasive, and easily applied in laboratories routine.

## Final considerations

**Figure 8:**
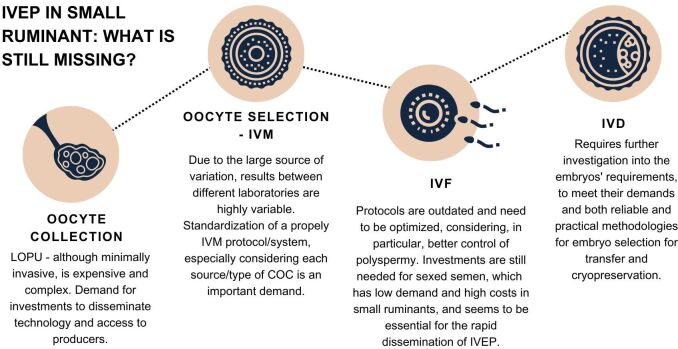
The schematic **figure ill**ustrates the main points to consider in each step of IVEP to disseminate this biotechnology in small ruminants. IVEP (*in vitro* embryo production), IVM (*in vitro* maturation), IVF (*in vitro* fertilization), and IVD (*in vitro* development).

The limited demand generates lower number of technicians and researchers involved in the IVEP industry in small ruminants, making its progress slower than in cattle and its application still restricted in these animals. To widespread IVEP in goats and sheep it is important to consider important aspects, such as: seasonality and the application of strategies to mitigate the effect of season; the need for simplification of oocyte collection from live females that is still complex compared to cattle; the demand for a standardization of the hormonal stimulatory and IVM protocols; the access to greater availability of sexed semen; and even, the identification of more reliable and noninvasive techniques for the selection of embryos for transfer and cryopreservation (Figure 8). Considering that, in general, IVEP outcomes are similar compared to cattle, the compilation of points that are still missing in each step of this biotechnology for its own optimization can be crucial and should be considered in research for a greater diffusion of this technique in small ruminants.
